# Sex-specific differences in the standard of care for infrarenal abdominal aortic aneurysm repair, and risk of major adverse cardiovascular events and death

**DOI:** 10.1093/bjs/znad018

**Published:** 2023-01-31

**Authors:** Anna L Pouncey, Michael J Sweeting, Colin Bicknell, Janet T Powell

**Affiliations:** Department of Surgery and Cancer, Imperial College London, London, UK; Department of Health Sciences, University of Leicester, Leicester, UK; Department of Surgery and Cancer, Imperial College London, London, UK; Department of Surgery and Cancer, Imperial College London, London, UK

## Abstract

**Background:**

This study investigated whether sex-specific differences in preoperative/perioperative standard of care (SOC) account for disparity in outcomes after elective infrarenal abdominal aortic aneurysm repair.

**Methods:**

This was a retrospective cohort study of elective infrarenal abdominal aortic aneurysm repairs (2013–2020) using depersonalized patient-level National Vascular Registry data. SOC was defined for waiting times, preoperative assessment (multidisciplinary/anaesthetic review), cardiovascular risk prevention, and perioperative medication. The primary outcome was major cardiovascular event and/or death (MACED).

**Results:**

Some 21 810 patients with an infrarenal abdominal aortic aneurysm were included, 2380 women and 19 430 men. Women less often underwent aneurysm repair within SOC waiting times (51.5 *versus* 59.3 per cent; *P* < 0.001), but were equally likely to receive preoperative assessment (72.1 *versus* 72.5 per cent; *P* = 0.742). Women were less likely to receive secondary prevention for known cardiac disease (34.9 *versus* 39.6 per cent; *P* = 0.015), but more often met overall cardiovascular risk prevention standards (52.1 *versus* 47.3 per cent; *P* < 0.001). Women were at greater risk of MACED (open: 12.0 *versus* 8.9 per cent, *P* < 0.001; endovascular: 4.9 *versus* 2.9 per cent, *P* < 0.001; risk-adjusted OR 1.33, 95 per cent c.i. 1.12 to 1.59). A significant reduction in the odds of MACED was associated with preoperative assessment (OR 0.86, 0.75 to 0.98) and SOC waiting times (OR 0.78, 0.69 to 0.87). There was insufficient evidence to confirm a significant sex-specific difference in the effect of SOC preoperative assessment (women: OR 0.69, 0.50 to 0.97; men: OR 0.89, 0.77 to 1.03; interaction *P* = 0.170) or SOC waiting times (women: OR 0.84, 0.62 to 1.16; men: OR 0.76, 0.67 to 0.87; interaction *P* = 0.570) on the risk of MACED.

**Conclusion:**

SOC waiting times and preoperative assessment were not met for both sexes, which was associated with an increased risk of MACED. Sex-specific differences in SOC attenuated but did not fully account for the increased risk of MACED in women.

## Teaser

This study used data from the National Vascular Registry (21 810 patients; 2380 women and 19 430 men) to investigate whether sex-specific differences in preoperative/perioperative standard of care account for disparity in major adverse cardiovascular event and/or death following elective infrarenal abdominal aortic aneurysm repair. The study demonstrated that provision of standard of care within the infrarenal abdominal aortic aneurysm repair pathway was associated with a reduction in the risk of major adverse cardiovascular event and/or death but was below minimum attainment targets for both sexes. Standard of care received was lower for women, and their risk of major adverse cardiovascular event and/or death was greater. Although standard of care did not fully account for the sex-specific disparity in outcomes, a significant risk reduction for women receiving preoperative assessment was observed.

## Introduction

Abdominal aortic aneurysm (AAA) repair presents a high-risk, low-volume procedure, with around 4000 elective repairs conducted in the UK per annum^1^. As such, it is difficult to identify quality of care on a service level, which may lead to false confidence in the standard of care (SOC) provided^2^. A successful aortic repair does not start and end at the operating table, as factors such as age and co-morbid status present a constellation of perioperative challenges which must be anticipated and addressed^3^. This issue is heightened for women, who are a minority within the AAA population, have a lower likelihood of receiving elective repair, a greater risk of rupture, are often older at the time of repair, and may have distinct care needs^4–6^.

The importance of coordinated, evidence-based care pathways is increasingly recognized^3,7^. Substantial change in the regulation of AAA repair in the UK was triggered when the 2008 VASCUNET report^8^ identified that UK mortality from open aortic aneurysm repair was the highest in Europe. A higher mortality risk for women (6.5 (95 per cent c.i. 5.2 to 8.0) per cent) compared with men (3.6 (3.2 to 4.0) per cent) was also observed^9^. In response, a national AAA quality improvement programme (AAAQIP) was established, aiming to standardize and improve patient care and outcomes, with high-quality audit via the National Vascular Registry (NVR)^10^.

Within this quality improvement framework, the importance of specialist anaesthetic assessment was recognized, and multidisciplinary team meetings (MDT) were emphasized to enable informed decision-making, with risk stratification, preoperative cardiovascular risk management, and appropriate surgical selection^10,11^. The efficiency and quality of service in the UK is monitored using waiting time standards, which have been scrutinized by the NVR and the Screening Quality Assurance Service since 2013^1,12^.

Cardiovascular risk stratification and management is of particular importance, as cardiac disease is manifest in approximately 36 per cent or more of the AAA population and, in the event of a postoperative myocardial infarction, a 41.2 per cent 1-year mortality rate is observed^13–16^. The National Institute for Health and Care Excellence (NICE) recommend that patients should be counselled on their risk of concomitant cardiovascular disease, and how this can be reduced through smoking cessation, lifestyle modification, optimization of medicines, and statin therapy^17–20^.

Following the implementation of AAAQIP and with increased uptake of endovascular aortic repair (EVAR), UK perioperative mortality rates significantly reduced, with contribution from widespread implementation of the National AAA Screening Programme (NAAASP) for men^21,22^. These changes have largely been hailed as a success. However, there remains a significantly higher risk of death and complications for women compared with men. A recent meta-analysis^4^ of elective AAA repair identified an increased odds of death for women for both open aortic repair (OAR) and EVAR, in the UK and worldwide. This sex-specific disparity in mortality risk persisted following multivariable risk adjustment for age and co-morbid status, and has not diminished with time or with the implementation of the AAAQIP^4^. Moreover, even though fewer women were diagnosed with cardiac disease before surgery, they were observed to have a similar (for OAR) or increased (for EVAR) risk of postoperative cardiac complications. They were also at increased risk of bowel ischaemia and lower limb ischaemia after EVAR^4^.

The aim of this study was to test the hypothesis that sex-specific differences in the preoperative and perioperative SOC may account for the disparity in major cardiovascular event and/or death (MACED) after elective infrarenal AAA repair.

## Methods

This retrospective cohort study used depersonalized patient-level electronic healthcare data from the NVR. Analysis of data was conducted following Healthcare Quality Improvement Partnership (HQIP) approval, under the General Data Protection Regulation Articles 6(1)(e) and 9(2)(j). Depersonalized data were stored and analysed in the Imperial Big Data Analytics Unit Secure Environment (BDAU SE) (Organization Data Service code EE133886-BDAU), which is ISO 27001-certified (Alcumus ISOQAR; certificate number 15484_ISN_001) and compliant with the NHS Digital Data Security and Protection Toolkit (organization code EE133887-BDAU). All users of the BDAU SE are bound to the Data Protection Act (DPA) by Imperial College DPA registration (registration number Z5940050). Reporting was conducted in accordance with STROBE guidance (http://www.strobe-statement.org/).

The study included all patients registered to the NVR between 1 January 2013 and 1 January 2020, who had undergone a primary infrarenal open or endovascular AAA repair. Ruptured or secondary AAA repair, thoracic or suprarenal aneurysms and aortic dissection were excluded from the analysis (*Fig. S1*).

### Standard-of-care targets

SOC variables were defined in accordance with the AAAQIP preoperative care bundle^10^, and secondary prevention (for example antiplatelet, antihypertensives, statins) in accordance with NICE, European Society for Vascular Surgery, and European Society of Cardiology best practice guidance standards^17–19^: SOC preoperative assessment—all patients should receive an MDT discussion and consultant anaesthetist review^10^; SOC waiting time—referral at threshold AAA size for treatment, measured in this study as time from first MDT discussion to operation, must be less than 2 weeks if symptomatic, or 8 weeks if asymptomatic, with minimum expected attainment of at least 60 per cent, and an achievable target of at least 80 per cent^12,17,23^; SOC preoperative medications (cardiovascular risk prevention)—all patients with an AAA should be prescribed a statin, all patients with a cardiac history must be prescribed an antiplatelet (or form of anticoagulation) and angiotensin-converting enzyme inhibitor/angiotensin receptor blocker (ACEi/ARB), in addition to a statin (secondary cardiac prevention), and all patients with a history of symptomatic peripheral arterial disease (PAD) must be prescribed an antiplatelet (or form of anticoagulation) in addition to a statin^17–19,24^; and SOC perioperative medications—all patients must receive antibiotic and deep venous thrombosis prophylaxis^24^. Where the minimum expected attainment level was not prespecified, this was assumed to be at least 80 per cent.

### Clinical outcomes

The primary outcome measure was the composite of major adverse cardiovascular event and/or (in-hospital/30-day) death (MACED). Major adverse cardiovascular event (MACE) was defined as cardiac complication, stroke, bowel ischaemia, limb ischaemia or paraplegia.

### Data quality and handling, and statistical analysis

Assessment of data quality was conducted in accordance with published guidance (*Appendix S1*)^25^. Clinical data are collected by vascular surgeons, which is then subject to a range of validation processes (https://www.vsqip.org.uk/surgeon-outcomes/explanatory-notes/). All pre-existing variables used are defined in the NVR data dictionary (https://www.vsqip.org.uk/resources/guides/nvr-data-dictionary/). Variables or patient records with insufficient data quality (defined as more than 50 per cent missing data), including patient frailty score, were excluded. Before multivariable regression, multiple imputation, using the k-nearest neighbours approach, was employed with comparison of source and imputed data sets for verification of acceptability (*Appendix S2*)^26,27^. Analyses were conducted according to a prespecified plan in R statistical software version 4.1.3 (R Foundation for Statistical Computing, Vienna, Austria); a list of packages used can be found in the *supplementary material*^28,29^. Sex-specific differences in preoperative status and receipt of standard of care, as well as unadjusted outcomes, are described using standard descriptive statistics, dependent on the category and distribution of data, and stratified by AAA repair type to account for patient selection and procedure-associated risk^25^. Logistic regression was used to assess trend in SOC attainment over time, adjusting for sex and repair category.

#### Analysis of sex-specific risk of MACED

For analysis of sex-specific risk of MACED, sex was treated as a relevant exposure, with analyses adjusted for the presence of relevant confounders. Univariate analyses, assessment of collinearity, and expert prioritization according to clinical relevance were conducted to select 26 variables: patient sex, age (less than 75 years, or older), repair type, sociodemographic factors (UK deprivation quintile and smoking status), co-morbid status (ischaemic heart disease, congestive heart failure, PAD, chronic kidney disease (defined by clinically recorded diagnosis), stroke, cancer, hypoalbuminaemia (below 35 g/dl), anaemia (less than 13 g/dl for men and less than 12 g/dl for women), abnormal ECG (assessed by anaesthetist before AAA repair), ASA grade), preoperative medications (anticoagulation, beta-blocker, and statin), preoperative SOC (preoperative assessment, waiting time, cardiovascular risk prevention, need for medication adjustment, and specialty referral), and AAA repair factors (aortic size index (ASI, calculated as aortic diameter/body surface area), symptomatic status, and general anaesthetic)^30^. Forwards stepwise inclusion of variables to build a full logistic regression model was first performed^27^. Backwards stepwise selection, using variable importance (regression coefficient) and statistical significance (*P* value), was then used to elucidate an optimal model, as specified by optimization of the Akaike information criteria^31^. To reduce the risk of overfitting, the data were split into training (90 per cent) and testing (10 per cent) data sets, stratified by sex and MACED, and a resampling scheme (k-fold cross-validation with 10 folds, repeated 5 times) was used on training data, with evaluation of the final model performance using the test set^27,31^.

### Analysis of sex-specific risk reduction associated with receipt of standard of care

For analysis of sex-specific risk reduction in MACED with receipt of SOC, SOC was treated as the relevant exposure, with sex as an effect modifier. Sex-specific interaction with SOC variables was introduced and evaluated in the optimal model, using the framework for analysis of interactions, introduced by Wu and Hamada^32^, which rests on three key principles: hierarchy, effect sparsity, and heredity. To comply with these principles, analyses were limited to two-way interactions, using only SOC variables demonstrated to have a significant effect on the risk of MACED in univariate analysis^32^. Marginal effects were then calculated to demonstrate the impact of patient sex on SOC-associated reduction in risk of MACED.

## Results

Some 21 810 patients (19 430 men and 2380 women) underwent elective repair of an infrarenal AAA; 6826 men and 841 women received OAR, whereas 12 604 men (64.9 per cent) and 1539 women (64.6 per cent) had EVAR (*Fig. S1*). A slight reduction in the proportion of AAAs treated with EVAR was observed from 2016 to 2019, but did not significantly differ for men and women (*supplementary material*).

### Preoperative cardiovascular status

Women were on average 2.5 years older and less likely to have a diagnosis of ischaemic heart disease (29.0 per cent (691 of 2380) *versus* 37.7 per cent (7326 of 19 430); *P* < 0.001), or abnormal ECG (28.1 per cent (667 of 2380) *versus* 32.8 per cent (6362 of 19 430); *P* < 0.001). No difference in congestive cardiac failure, stroke, PAD or anaemia were observed. Women were more frequently from a lower UK deprivation decile (*P* < 0.001) and more likely to be a current smoker (26.1 per cent (622 of 2380) *versus* 21.4 per cent (4160 of 19 430); *P* < 0.001).

Overall, women were less likely to receive prescription of a statin (77.1 per cent (1834 of 2380) *versus* 80.6 per cent (15 652 of 19 430); *P* < 0.001), antiplatelet (72.2 per cent (1719 of 2380) *versus* 75.2 per cent (14 620 of 19 430); *P* = 0.006), beta-blocker (28.4 per cent (675 of 2380) *versus* 30.6 per cent (5940 of 19 430); *P* = 0.029) or ACEi/ARB (34.5 per cent (821 of 2380) *versus* 38.1 per cent (7410 of 19 430); *P* = 0.001).

On average, women were treated at a marginally smaller AAA diameter (mean(s.d.) 60.13 (9.01) *versus* 61.62 (10.16) mm; *P* < 0.001) but this equated to a greater difference in ASI (3.46 (0.69) *versus* 3.07 (0.62); *P* < 0.001). Women were also slightly more likely to have a symptomatic aneurysm at the time of repair (5.5 per cent (132 of 2380) *versus* 3.3 per cent (641 of 19 430); *P* < 0.001), perhaps reflecting the influence of the NAAASP. Further sex-specific differences in demographics, risk factors, and investigations are provided in *Table S1*.

### Receipt of standard of care

**Fig. 1:**
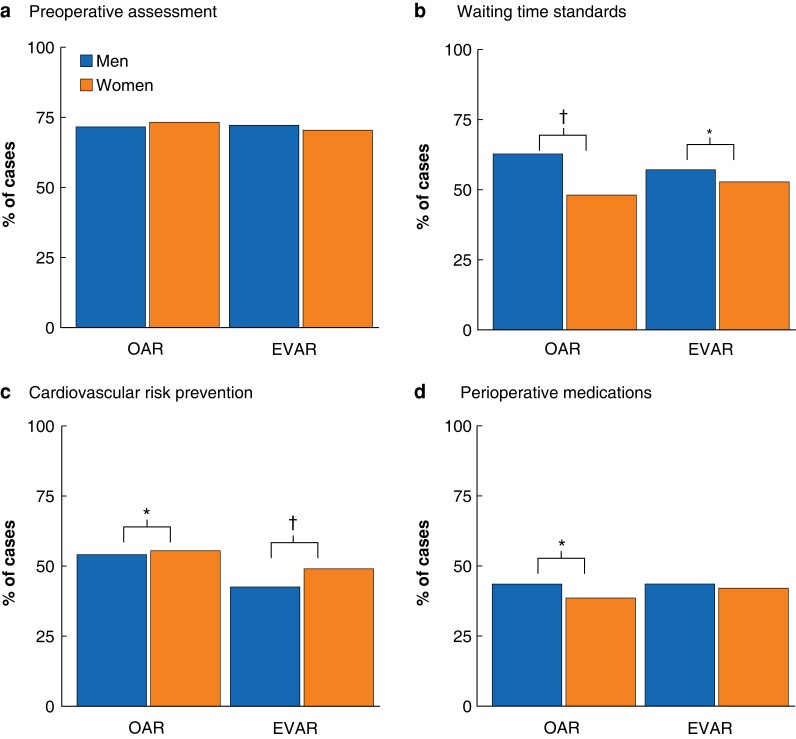
Percentage of men and women undergoing elective infrarenal abdominal aortic aneurysm repair, treated according to standard of care **a** Preoperative assessment, **b** treatment within waiting time standards, **c** cardiovascular risk prevention, and **d** perioperative medications (antibiotic and deep venous thrombosis prophylaxis). OAR, open aortic repair; EVAR, endovascular aortic repair. *P < 0.010, †*P* < 0.001 (Chi-square test).

Reported receipt of all SOC variables was deficient for both sexes (*Fig. 1* and *Table S2*). Women and men were equally likely to receive SOC preoperative assessment (MDT discussion and consultant anaesthetist review) for both open and endovascular repair (overall: 72.1 per cent (1717 of 2380) *versus* 72.5 per cent (14 084 of 19 430), *P* = 0.742; OAR: 72.9 per cent (613 of 841) *versus* 71.6 per cent (4887 of 6826), *P* = 0.455; EVAR: 71.7 per cent (1104 of 1539) *versus* 73.0 per cent (9197 of 12 604), *P* = 0.319). Slight improvement in SOC attainment over time was observed (OR 1.07, 95 per cent c.i. 1.06 to 1.09; *P* < 0.001), but levels remained below minimum attainment standards (2019—OAR: 74.8 per cent (98 of 131) *versus* 76.2 per cent (825 of 1083), *P* = 0.729; EVAR: 79.5 per cent (136 of 171) *versus* 76.4 per cent (1176 of 1539), *P* = 0.360) (*supplementary material*).

Women were less likely to receive AAA repair within SOC waiting time (51.5 per cent (983 of 1909) *versus* 59.3 per cent (9279 of 15 645); *P* < 0.001). This difference was more pronounced for OAR (48.2 per cent (327 of 679) *versus* 62.9 per cent (3460 of 5505); *P* < 0.001) than EVAR (53.3 per cent (656 of 1230) *versus* 57.9 per cent (5819 of 10 051); *P* = 0.003) and did not change over time (*P* = 0.152) (*Fig. 1*, *Table S2*, and *supplementary material*).

In accordance with preoperative diagnosis of cardiovascular co-morbidity, women were more likely to meet SOC cardiovascular risk prevention criteria (52.1 (1241 of 2830) *versus* 47.3 per cent (9190 of 19 430); *P* < 0.001). The difference was more pronounced for EVAR (50.3 per cent (774 of 1539) *versus* 43.8 per cent (5521 of 12 604); *P* < 0.001) than OAR (55.5 per cent (467 of 841) *versus* 53.8 per cent (3669 of 6826); *P* = 0.001) and did not change over time (*P* = 0.392). However, this may reflect the lower incidence of preoperative cardiovascular disease, as, in subgroup analysis of patients with known cardiac disease, women were less likely to receive secondary preventative medications (34.9 per cent (241 of 691) *versus* 39.6 per cent (2902 of 7326); *P* = 0.015), with no evidence of a change over time (*P* = 0.440) (*Fig. 1*, *Table S2*, and *supplementary material*).

Women were also less likely to have NVR-recorded receipt of SOC perioperative medications (deep venous thrombosis and antibiotic prophylaxis) for OAR (38.5 per cent (324 of 841) *versus* 43.2 per cent (2952 of 6826); *P* = 0.010) but not EVAR (42.4 per cent (653 of 1539) *versus* 43.2 per cent (5440 of 12 604); *P* = 0.604). Over time, a significant improvement in recorded SOC was observed (OR 1.86, 1.82 to 1.90; *P* < 0.001), but levels remained below guidance standard attainment (2019—OAR: 54.2 per cent (71 of 131) *versus* 63.1 per cent (683 of 1083), *P* = 0.048; EVAR: 58.5 per cent (100 of 171) *versus* 59.5 per cent (916 of 1539), *P* = 0.793) (*Fig. 1*, *Table S2*, and *supplementary material*).

### Risk of MACED

**Fig. 2:**
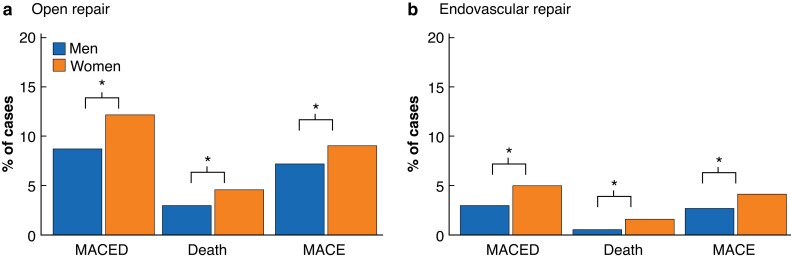
Percentage of men and women experiencing a perioperative major adverse cardiovascular event or death after infrarenal abdominal aortic aneurysm repair **a** Open and **b** endovascular aortic repair. MACED, major adverse cardiovascular event or death; MACE, major adverse cardiovascular event. *P < 0.001 (Chi-square test).

Women were at greater risk of MACED after OAR (12.0 per cent (101 of 841) *versus* 8.9 per cent (612 of 6826); OR 1.38, 95 per cent c.i. 1.10 to 1.73; *P* < 0.001) and EVAR (4.9 per cent (76 of 1539) *versus* 2.9 per cent (370 of 12 604); OR 1.71, 1.33 to 2.21; *P* < 0.001). In-hospital/30-day mortality was greater for women for both OAR (4.4 per cent (37 of 841) *versus* 2.8 per cent (194 of 6826); OR 1.57, 1.10 to 2.25; *P* = 0.017) and EVAR (1.5 per cent (23 of 1539) *versus* 0.5 per cent (62 of 12 604); OR 3.07, 1.89 to 4.97; *P* < 0.001), and the overall incidence of MACE was greater for women than men for both OAR (8.9 per cent (69 of 773) *versus* 7.2 per cent (465 of 6491); OR 1.30, 1.00 to 1.70; *P* < 0.001) and EVAR (4.0 per cent (76 of 1539) *versus* 2.6 per cent (370 of 12 523); OR 1.57, 1.18 to 2.07; *P* < 0.001) (*Fig. 2* and *Table S3*).

#### Sex-specific risk of MACED

**Fig. 3:**
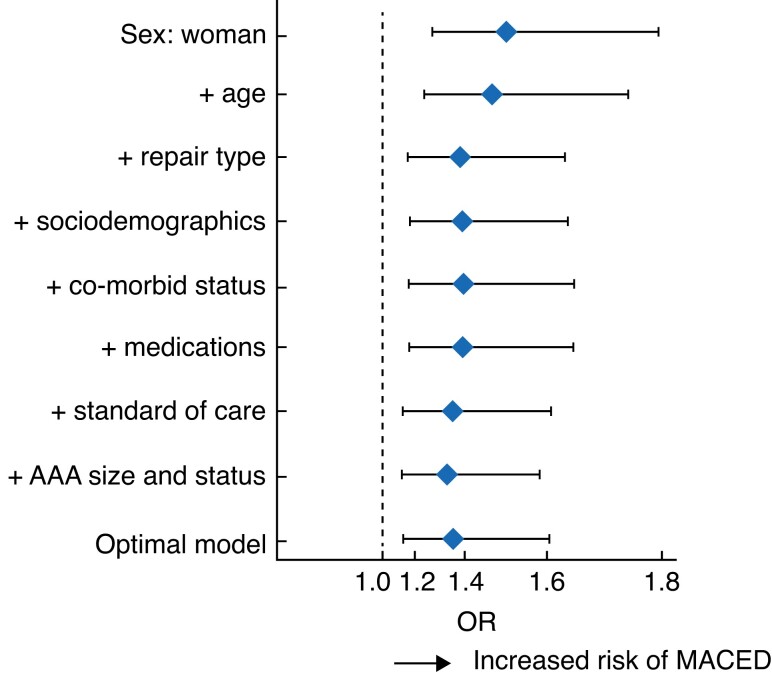
Sex-specific odds of major adverse cardiovascular event or death Unadjusted odds for women are shown, followed by those for forwards stepwise models adjusting for age (≥ 75 years), repair type (endovascular repair), sociodemographic factors (UK deprivation quintile and smoking status), co-morbid status (ischaemic heart disease, congestive heart failure, peripheral arterial disease, chronic kidney disease, stroke, cancer, hypoalbuminaemia (below 35 g/dl), anaemia (less than 13 g/dl for men and less than12 g/dl for women), abnormal ECG, ASA grade), medications (anticoagulation, beta-blocker, and statin), preoperative standard of care (preoperative assessment, treatment within waiting time targets, cardiovascular risk prevention, need for medication adjustment, and specialty referral), and abdominal aortic aneurysm (AAA) repair factors (aortic size index, symptomatic status, and general anaesthetic), and for the optimal logistic regression model selected by backwards selection. ORs are shown with 95% confidence intervals. MACED, major adverse cardiovascular event or death.

Following progressive adjustment for selected variables (age, repair type, sociodemographic factors, co-morbid status, medications, preoperative SOC, and AAA repair factors), the effect of sex on the risk of MACED was attenuated (OR 1.51, 95 per cent c.i. 1.27 to 1.78, *P* < 0.001 to OR 1.33, 1.12 to 1.59, *P* < 0.001), but remained significant (*Fig. 3* and *Table S4*).

**Fig. 4:**
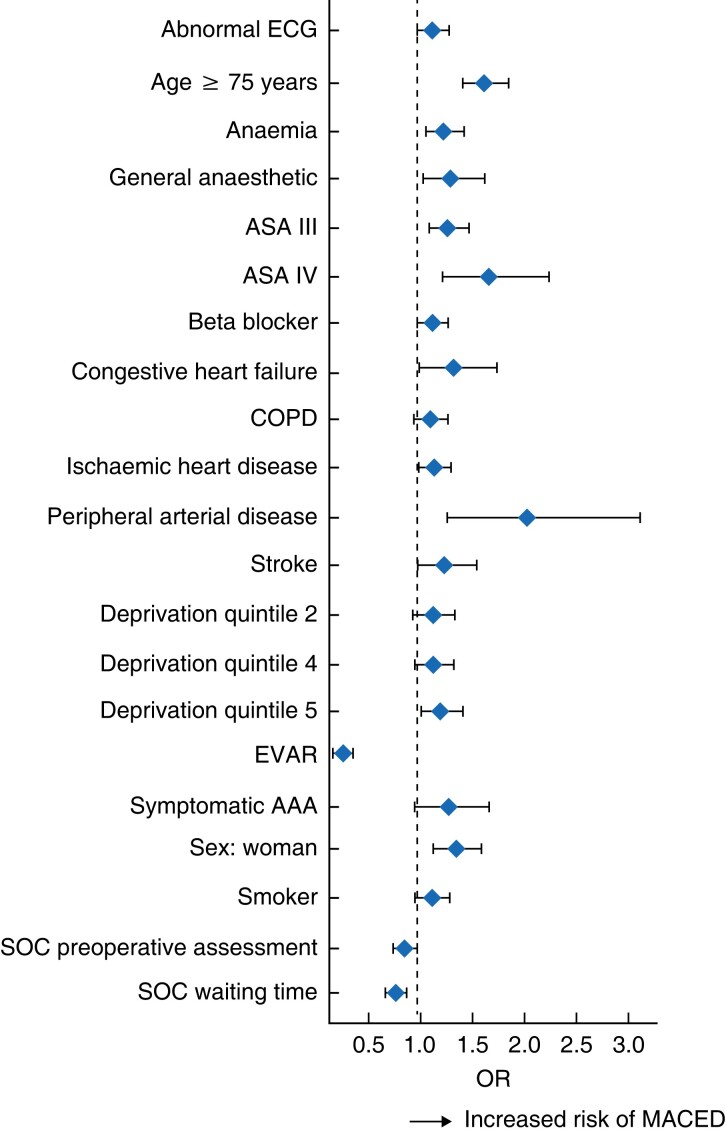
Optimal logistic regression model obtained by backwards selection ORs are shown with 95% confidence intervals. Reference categories: ASA I, deprivation quintile 1. COPD, chronic obstructive pulmonary disease; EVAR, endovascular aortic repair; SOC, standard of care; MACED, major adverse cardiovascular event or death.

Adjustment for operative modality (open *versus* endovascular) was an important factor in this attenuation of risk. A minor attenuation of the effect of sex on the risk of MACED was observed after additional adjustment for SOC variables from an OR of 1.39 (1.17 to 1.65; *P* < 0.001) to 1.36 (1.14 to 1.62; *P* < 0.001). As demonstrated in the optimal model, a reduction in risk of MACED was also observed with use of EVAR (OR 0.26, 0.23 to 0.30; *P* < 0.001), whereas an increase in the odds of MACED was observed for use of general anaesthetic (OR 1.30, 1.05 to 1.63; *P* = 0.017), age 75 years or more (OR 1.63, 1.43 to 1.86; *P* < 0.001), and for patients with PAD (OR 2.04, 1.31 to 3.19; *P* = 0.002), ASA grade III (OR 1.27, 1.24 to 1.48; *P* = 0.002) or IV (OR 1.67, 1.24 to 2.25; *P* < 0.001) and anaemia (OR 1.24, 1.06 to 1.44; *P* = 0.005) (*Fig. 4* and *Table S5*).

#### Sex-specific reduction in risk of MACED associated with standard of care

Overall, a significant reduction in the odds of MACED was associated with receipt of SOC preoperative assessment (OR 0.86, 95 per cent c.i. 0.75 to 0.98; *P* = 0.021) and treatment within SOC waiting times (OR 0.78, 0.69 to 0.87; *P* < 0.001) (*Fig. 4* and *Table S6*). SOC cardiovascular risk prevention (OR 0.93, 0.83 to 1.06; *P* = 0.276) and SOC perioperative medication (OR 0.98, 0.87 to 1.11; *P* = 0.766) did not significantly reduce the risk of MACED and did not contribute to the model.

**Fig. 5:**
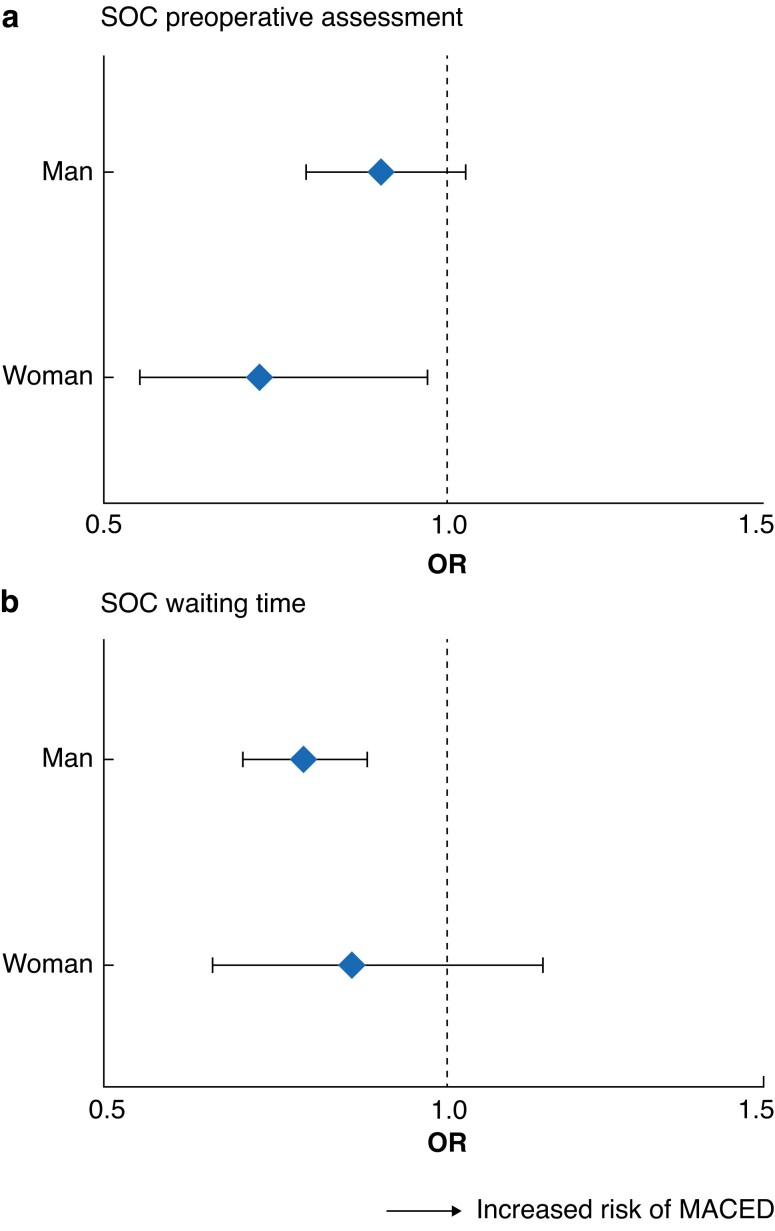
Sex-specific reduction in odds of major adverse cardiovascular event or death associated with standard of care **a** Standard-of-care (SOC) preoperative assessment and **b** SOC waiting times. ORs are shown with 95% confidence intervals. MACED, major adverse cardiovascular event or death.

Although there was insufficient evidence to confirm any sex-specific differences in the effect of SOC preoperative assessment (interaction *P* = 0.175), on resolution of interaction terms, a significant reduction in risk of MACED was observed for women only (women: OR 0.69, 0.50 to 0.97, *P* = 0.026; men: OR 0.89, 0.77 to 1.03; *P* = 0.106). Conversely, although there was insufficient evidence to confirm any sex-specific differences in the effect of SOC waiting time (interaction *P* = 0.555), on resolution of interaction terms, a significant reduction in the risk of MACED was noted for men only (women: OR 0.84, 0.62 to 1.16, *P* = 0.354; men: OR 0.76, 0.67 to 0.87, *P* < 0.001) (*Fig. 5* and *Table S6*).

## Discussion

SOC targets were not met for both women and men for OAR and EVAR. Although a slight improvement in the recorded rates of preoperative assessment and perioperative medications was observed over time, all metrics remained below the minimum expected attainment levels (apart from OAR waiting times for men, achieved in 62.9 per cent)^23^. Preoperative assessment and treatment within waiting times were associated with a significant reduction in the risk of MACED, and these data suggest that efforts to improve preoperative care may reduce risk for both women and men.

Women were at greater risk of MACED after both OAR and EVAR, and an inferior rate of SOC attainment was observed for women with regard to waiting times, receipt of perioperative medications, and secondary cardiac prevention. Despite these differences, adjustment for SOC only mildly attenuated the sex-specific disparity in MACED (OR 1.33, 95 per cent c.i. 1.12 to 1.59); these data suggested, but did not confirm, the presence of any significant sex-specific difference in SOC efficacy.

As with all registry healthcare data, this study is subject to quality of data entry and clinician reporting and, as such, the SOC variables serve as a proxy for overall SOC. The reduction in risk of MACED associated with preoperative assessment, particularly for women, may reflect the importance of preoperative optimization with specialist multidisciplinary (for example anaesthetic and medical) input, and appropriate risk stratification and selection for repair, as previously reported by Howell^20^ and others^3,33^ in the UK. Treatment within waiting time targets occurred more often in men than women, and led to a significant reduction in the risk of MACED for men. This may reflect optimal preoperative physiological or anatomical status (beyond the standard NVR indicators used) in those who met waiting time targets compared with those requiring additional preoperative investigation and intervention; alternatively, prolonged waiting times may indicate strain on healthcare services to the detriment of SOC^34^.

The benefit of cardiovascular risk stratification and treatment for patients with an AAA is well known. Use of a statin and aspirin are shown to reduce the odds of death, whereas smoking and uncorrected coronary disease increase the risk of MACED after AAA repair^35^. However, in this study, SOC cardiovascular preventative treatment did not seem to significantly reduce the risk of MACED. This may reflect poor adherence to medication, as approximately 52 per cent of patients under AAA surveillance have been shown to be non-compliant with use of statins, antiplatelets or smoking cessation^36,37^; or inadequacies in diagnosis and provision of preventative treatment^14,37^. Moreover, a major weakness of these data is that the intensity of statin therapy and reduction in low density lipoprotein–cholesterol levels, which directly correlate with a reduction in the risk of major vascular events and mortality^38,39^, are not described. This warrants further investigation.

Diagnostic discordance was observed for women, who were less likely to have known concomitant cardiovascular disease before operation or to receive secondary cardiac prevention, but had a higher risk of postoperative MACE. This may arise because of sex-specific differences in the efficacy of clinical care. It has been reported that women with cardiac disease are less likely to be investigated, or to receive standard treatment in line with guidance, which can result in a care deficit and excess mortality^40,41^. Routine testing for cardiac disease is also less sensitive for women, in whom non-obstructive disease is more common^42–44^. Alternatively, the increased risk of MACE could also arise from other less explored factors, such as sex-specific difference in thromboembolic risk^45^. As such, further detailed assessment is needed to establish contributing factors, and whether sex-specific cardiovascular risk assessment might carry benefit for women undergoing AAA repair^46^.

This study used data from the NVR, which comprises a valuable resource enabling analysis of clinical practice on a national scale^1^. As such, this study presents a detailed analysis of short-term infrarenal AAA repair outcomes and SOC in a large cohort. Findings from these data are applicable to all practice in the UK, but require external corroboration in other countries and healthcare systems. These data also reflect AAA repair before the coronavirus (COVID-19) pandemic, which had a dramatic and detrimental impact on service provision^47,48^.

As a retrospective observational study of real-world practice, this work is subject to unavoidable systemic sex-specific bias in AAA detection, introduced by the NAAASP, and in surgical selection, with analyses limited to patients who underwent AAA repair. To reduce heterogeneity in clinical status and anatomical complexity, the analysis was restricted to elective repair of infrarenal AAA, stratified by symptomatic status, which influences the time for preoperative assessment and treatment owing to the urgency of the procedure. Patients identified by the NAAASP were not noted to have a greater likelihood of SOC cardiovascular risk prevention than those identified outside of screening; they were not included as a further subgroup in this analysis because relevant confounders that may reflect earlier detection (such as age, co-morbid status, and ASI) had already been accounted for.

Analyses were also constrained to variables recorded in the NVR. As a result, the time frame of the reported outcome (MACED) was restricted to in-hospital-to-30-day events. This does not encompass all relevant postoperative MACED which occurs up to 90 days and beyond^49^. The rate of attainment for SOC perioperative medications was also lower than expected and raises concern regarding either the SOC provided or the accuracy of these data. To account for these factors, further validation of data quality, with detailed assessment of preoperative pathways, as well as analyses of long-term outcomes, will be conducted in the future using healthcare data linkage from an alternative source.

Optimization of preoperative and perioperative care during AAA repair to both improve outcomes and the patient journey is an evolving and increasingly complex field. Indeed, the Society for Vascular Surgery and Enhanced Recovery After Surgery Society recently provided 36 recommendations, of which ‘cardiac risk should be evaluated and optimized…’ is but one^3^. However, caution should be taken as care pathways develop to monitor attainment and patient outcomes. This study raises major concern that, in the UK, core SOC targets for AAA are not being met and that these are associated with an increased risk of MACED. This has significant implications for men, in whom the majority of AAA repairs are carried out, and for women, who were overall less likely to receive SOC. First and foremost, investigation into the accuracy of reporting and barriers to SOC attainment must be conducted to improve care for both sexes. Second, further investigation and reporting of health disparities should be encouraged to enable future care pathways to develop in a framework conducive to health equity. Only then can health injustices, including sex-specific disparities in AAA repair outcomes, be addressed^50,51^.

## Supplementary Material

znad018_Supplementary_Data

## Data Availability

Relevant data from the NVR are available on application to the HQIP.
