# Supplementary material for: Sex-specific differences in the standard of care for infrarenal abdominal aortic aneurysm repair, and risk of major adverse cardiovascular events and death
Source: Br J Surg. 2023 Jan 31;110(4):481–8. doi: 10.1093/bjs/znad018 (PMC10364552; doi:10.1093/bjs/znad018)
Supplement: znad018_Supplementary_Data [file znad018_supplementary_data.docx]

Sex Specific Differences in the Standard of Care for Infrarenal Abdominal Aortic Aneurysm Repair, and Risk of Major Adverse Cardiovascular Events and Death.

Short Title: Sex-specific Differences in Standard of Care and Outcomes for AAA repair.

Anna L Pouncey1, BA (Hons.), BM BCh, MRCS (Eng.)

Guy Martin1, MBBS PhD MRCS

Colin Bicknell1, BM, MD, FRCS

Michael J Sweeting^2^, BSc, PhD

Janet T Powell1, MD, PhD

1. Department of Surgery and Cancer, Imperial College London, UK

2. Department of Health Sciences, University of Leicester, Leicester, UK

Correspondence to: Miss Anna Pouncey
Address: Department of Surgery and Cancer, QEQM, St Mary’s Hospital, Imperial College NHS Trust, Praed Street, London, W2 1NY
Email: anna-louise.pouncey@nhs.net

ORCID ID: 0000-0003-2329-6193

Twitter: @pounce321

**Supplementary Materials - Index**

| **Supplementary Methods** |  |
| --- | --- |
| List of R packages utilised | *pag. 2* |
| **Supplementary Results** |  |
| Percentage of endovascular cases, per annum | *pag. 3* |
| Trends in standard of care (SOC) targets attainment over time | *pag. 4-5* |
| **Supplementary Figures and Tables** |  |
| Figure S1. Flow chart demonstrating selection of elective infrarenal abdominal aortic aneurysm cases from the National Vascular Registry database. | *pag. 6* |
| Table S1. Sex-specific differences in demographics, risk factors and investigations prior to IRAAA repair. | *pag. 7-8* |
| **Table S2.** Sex-specific differences in receipt of pre-operative assessment and standard of care. | *pag. 9* |
| **Table S3.** Sex-specific differences in post-operative complications | *pag. 10* |
| Table S4. Sex specific odds of MACED | *pag. 11* |
| Table S5. ‘Optimal’ logistic regression model selected by backwards selection. | *pag. 12* |
| **Table S6.** Sex-specific effect of standard of care on risk of MACED. | *pag. 13* |
| **Supplementary Appendixes** |  |
| **Appendix S1.** Summary of missing data | *pag. 14* |
| **Appendix S2.** Summary of K-nearest neighbours’ multiple imputation | *pag. 15-16* |
| **References** | *pag. 17* |

**Supplementary Methods**

List of R packages utilised:

library(dplyr)

library(tidyverse)

library(tidymodels)

library(tidyr)

library(xlsx)

library(magrittr)

library(knitr)

library(ggplot2)

library(scales)

library(GGally)

library(themis)

library(naniar)

library(UpSetR)

library(car)

library(caret)

library(plotly)

library(utils)

library(pROC)

library(margins)

**Supplementary Results**

Percentage of elective endovascular infrarenal AAA repairs, for men and women, per annum.

|  | IR OAR | | IR EVAR | | |
| --- | --- | --- | --- | --- | --- |
|  | Men | Women | Men | Women | X^2^ |
|  | 6826 | 841 | 12604 | 1539 | p |
| Year, n (%) | | |  | | |
| 2014 | 1263 | 173 | 2329 (64.8) | 312 (64.3) | 0.826 |
| 2015 | 1138 | 151 | 2385 (67.7) | 284 (65.3) | 0.311 |
| 2016 | 1060 | 133 | 2348 (68.9) | 283 (68.0) | 0.718 |
| 2017 | 1125 | 136 | 2207 (66.2) | 258 (65.5) | 0.765 |
| 2018 | 1157 | 117 | 1796 (60.8) | 231 (66.4) | 0.044 |
| 2019 | 1083 | 131 | 2539 (58.7) | 171 (56.6) | 0.489 |

Logistic regression was utilised for assessment of trend in SOC attainment over time, adjusting for sex and repair category.

**SOC Pre-operative Assessment**

| Coefficients: | Odds Ratio | z value | p.value | Lower 95% Confidence Interval | Upper 95% Confidence Interval |
| --- | --- | --- | --- | --- | --- |
| Sex: Woman | 0.989 | -0.230 | 0.818 | 0.900 | 1.088 |
| Year | 1.074 | 7.865 | <0.001 | 1.055 | 1.094 |
| EVAR | 1.069 | 2.103 | 0.036 | 1.004 | 1.137 |
| Null deviance: 25678 on 21809 degrees of freedom Residual deviance: 25612 on 21806 degrees of freedom AIC: 25620 | | | | | |
|  |  |  |  |  |  |
|  |  |  |  |  |  |
|  |  |  |  |  |  |

**SOC Waiting Times**

| Coefficients: | Odds Ratio | z value | p.value | Lower 95% Confidence Interval | Upper 95% Confidence Interval |
| --- | --- | --- | --- | --- | --- |
| Sex: Woman | 0.717 | -6.840 | <0.001 | 0.652 | 0.789 |
| Year | 0.987 | -1.433 | 0.152 | 0.969 | 1.000 |
| EVAR | 0.850 | -5.014 | <0.001 | 0.798 | 0.906 |
| Null deviance: 25678 on 21809 degrees of freedom Residual deviance: 25612 on 21806 degrees of freedom AIC: 25620 | | | | | |
|  |  |  |  |  |  |
|  |  |  |  |  |  |
|  |  |  |  |  |  |

**SOC Cardiovascular Risk Prevention**

| Coefficients: | Odds Ratio | z value | p.value | Lower 95% Confidence Interval | Upper 95% Confidence Interval |
| --- | --- | --- | --- | --- | --- |
| Sex: Woman | 1.014 | 0.304 | 0.761 | 0.929 | 1.107 |
| Year | 1.016 | 1.895 | 0.058 | 0.999 | 1.033 |
| EVAR | 0.741 | -10.123 | <0.001 | 0.698 | 0.785 |
| Null deviance: 28949 on 21809 degrees of freedom Residual deviance: 28840 on 21806 degrees of freedom AIC: 28848 | | | | | |
|  |  |  |  |  |  |
|  |  |  |  |  |  |
|  |  |  |  |  |  |

**SOC Ischaemic Heart Disease Secondary Risk Prevention**

| Coefficients: | Odds Ratio | z value | p.value | Lower 95% Confidence Interval | Upper 95% Confidence Interval |
| --- | --- | --- | --- | --- | --- |
| Sex: Woman | 1.014 | 0.304 | 0.761 | 0.692 | 0.960 |
| Year | 1.016 | 1.895 | 0.058 | 0.989 | 1.044 |
| EVAR | 0.741 | -10.123 | <0.001 | 0.767 | 0.934 |
| Null deviance: 10737 on 8016 degrees of freedom Residual deviance: 10719 on 8013 degrees of freedom AIC: 10727 | | | | | |
|  |  |  |  |  |  |
|  |  |  |  |  |  |
|  |  |  |  |  |  |

**SOC Peri-operative Medications**

| Coefficients: | Odds Ratio | z value | p.value | Lower 95% Confidence Interval | Upper 95% Confidence Interval |
| --- | --- | --- | --- | --- | --- |
| Sex: Woman | 0.945 | -1.147 | 0.252 | 0.858 | 1.041 |
| Year | 1.858 | 61.528 | <0.001 | 1.822 | 1.895 |
| EVAR | 1.150 | 4.329 | <0.001 | 1.080 | 1.225 |
| Null deviance: 29801 on 21809 degrees of freedom Residual deviance: 25124 on 21806 degrees of freedom AIC: 25132 | | | | | |
|  |  |  |  |  |  |
|  |  |  |  |  |  |
|  |  |  |  |  |  |

**Supplementary Figures and Tables**

**Figure S1.** Flow chart demonstrating the selection of elective infrarenal abdominal aortic aneurysm cases from the NVR database. (All cases were confirmed to have >50% data capture.) *Abbreviations: OPCS – classification of interventions and procedures*

Table S1. Sex-specific differences in demographics, risk factors and investigations prior to IRAAA repair.

*Abbreviations:, ACEi/ARB – Angiotensin Converting Enzyme inhibitor/Angiotensin Receptor Blocker, American Society of Anaesthesiology, BMI – body mass index, CHF – Congestive Heart Failure, CKD – Chronic Kidney Disease, COPD – Chronic Obstructive Pulmonary Disease, ECG – Electrocardiogram, EVAR – Endovascular Aortic Repair, IHD – Ischaemic Heart Disease , IRAAA – Infrarenal Abdominal Aortic Aneurysm, OAR – Open Aortic Repair, p = p value, PAD – Peripheral Arterial Disease, SD – Standard Deviation)*

|  | All IRAAA | | | OAR | | | EVAR | | |
| --- | --- | --- | --- | --- | --- | --- | --- | --- | --- |
|  | Men | Women | P | Men | Women | p | Men | Women | p |
| n | 19430 | 2380 |  | 6826 | 841 |  | 12604 | 1539 |  |
| Co-morbid Status n (%) |  |  |  |  |  |  |  |  |  |
| Age mean (SD) | 74.24 (7.53) | 76.69 (7.20) | <0.001 | 70.65 (6.96) | 73.64 (6.99) | <0.001 | 76.18 (7.10) | 78.36 (6.75) | <0.001 |
| BMI mean (SD) | 28.88 (11.11) | 28.09 (19.74) | 0.003 | 28.79 (11.13) | 28.75 (30.93) | 0.940 | 28.92 (11.10) | 27.73 (8.98) | <0.001 |
| No Comorbidity | 2461/19430 (12.7) | 257/2380 (10.8) | 0.010 | 1160/6826 (17.0) | 109/841 (13.0) | 0.003 | 1301/12604 (10.3) | 148/1539 (9.6) | 0.414 |
| Diabetes | 3087/19430 (15.9) | 287/2380 (12.1) | <0.001 | 842/6826 (12.3) | 83/841 (9.9) | 0.044 | 2245/12604 (17.8) | 204/1539 (13.3) | <0.001 |
| Hypertension | 13278/19430 (68.3) | 1675/2380 (70.4) | 0.045 | 4511/6826 (66.1) | 575/841 (68.4) | 0.199 | 8767/12604 (69.6) | 1100/1539 (71.5) | 0.129 |
| COPD | 4839/19430 (24.9) | 767/2380 (32.2) | <0.001 | 1316/6826 (19.3) | 244/841 (29.0) | <0.001 | 3523/12604 (28.0) | 523/1539 (34.0) | <0.001 |
| IHD | 7326/19430 (37.7) | 691/2380 (29.0) | <0.001 | 2094/6826 (30.7) | 209/841 (24.9) | 0.001 | 5232/12604 (41.5) | 482/1539 (31.3) | <0.001 |
| CHF | 839/19430 (4.3) | 98/2380 (4.1) | 0.688 | 134/6826 (2.0) | 19/841 (2.3) | 0.654 | 705/12604 (5.6) | 79/1539 (5.1) | 0.493 |
| CKD | 2355/19430 (12.1) | 371/2380 (15.6) | <0.001 | 597/6826 (8.7) | 128/841 (15.2) | <0.001 | 1758/12604 (13.9) | 243/1539 (15.8) | 0.055 |
| Stroke | 1272/19430 (6.5) | 135/2380 (5.7) | 0.111 | 368/6826 (5.4) | 43/841 (5.1) | 0.797 | 904/12604 (7.2) | 92/1539 (6.0) | 0.094 |
| Cancer | 270/19430 (1.4) | 29/2380 (1.2) | 0.559 | 71/6826 (1.0) | 15/841 (1.8) | 0.079 | 199/12604 (1.6) | 14/1539 (0.9) | 0.054 |
| PAD | 179/19430 (0.9) | 22/2380 (0.9) | 1.000 | 70/6826 (1.0) | 9/841 (1.1) | 1.000 | 109/12604 (0.9) | 13/1539 (0.8) | 1.000 |
| Abnormal ECG | 6362/19405 (32.8) | 667/2371 (28.1) | <0.001 | 1733/6814 (25.4) | 192/836 (23.0) | 0.131 | 4629/12591 (36.8) | 475/1535 (30.9) | <0.001 |
| Anaemia | 3180/13709 (23.2) | 371/1636 (22.7) | 0.638 | 844/4826 (17.49) | 108/576 (18.75) | 0.453 | 2336/8883 (26.30) | 263/1060 (24.81) | 0.298 |
| Hypoalbuminaemia | 1762/13496 (13.06) | 268/1667 (16.09) | <0.001 | 700/4841 (14.46) | 108/606 (17.82) | 0.028 | 1062/8655 (12.27) | 160/1060 (15.09) | 0.009 |
| ASA Grade n (%) |  |  |  |  |  |  |  |  |  |
| 1 | 198 (1.0) | 24 (1.0) | <0.001 | 98 (1.4) | 11 (1.3) | <0.001 | 100 (0.8) | 13 (0.8) | <0.001 |
| 2 | 5438 (28.0) | 568 (23.9) |  | 2397 (35.1) | 234 (27.9) |  | 3041 (24.1) | 334 (21.7) |  |
| 3 | 13006 (67.0) | 1653 (69.5) |  | 4159 (61.0) | 556 (66.2) |  | 8847 (70.2) | 1097 (71.3) |  |
| 4 | 778 (4.0) | 132 (5.5) |  | 167 (2.4) | 39 (4.6) |  | 611 (4.8) | 93 (6.0) |  |
| 5 | 2 (0.0) | 2 (0.1) |  | 1 (0.0) | 0 (0.0) |  | 1 (0.0) | 2 (0.1) |  |
| *Missing Data* | *8/19430* | *0/2380* |  | *4/6826* | *0/841* |  | *4/12604* | *0/1539* |  |
| Deprivation Decile n (%) |  |  |  |  |  |  |  |  |  |
| 1 | 1177 (7.3) | 200 (10.4) | <0.001 | 443 (7.8) | 81 (11.9) | <0.001 | 734 (7.0) | 119 (9.7) | <0.001 |
| 2 | 1266 (7.8) | 197 (10.3) |  | 434 (7.7) | 75 (11.0) |  | 832 (7.9) | 122 (9.9) |  |
| 3 | 1466 (9.1) | 179 (9.4) |  | 514 (9.1) | 70 (10.3) |  | 952 (9.0) | 109 (8.8) |  |
| 4 | 1582 (9.8) | 200 (10.4) |  | 537 (9.5) | 66 (9.7) |  | 1045 (9.9) | 134 (10.9) |  |
| 5 | 1717 (10.6) | 199 (10.4) |  | 603 (10.7) | 70 (10.3) |  | 1114 (10.6) | 129 (10.5) |  |
| 6 | 1830 (11.3) | 229 (12.0) |  | 672 (11.9) | 84 (12.3) |  | 1158 (11.0) | 145 (11.8) |  |
| 7 | 1739 (10.7) | 181 (9.5) |  | 573 (10.1) | 65 (9.5) |  | 1166 (11.1) | 116 (9.4) |  |
| 8 | 1886 (11.6) | 208 (10.9) |  | 682 (12.1) | 68 (10.0) |  | 1204 (11.4) | 140 (11.4) |  |
| 9 | 1814 (11.2) | 162 (8.5) |  | 601 (10.6) | 54 (7.9) |  | 1213 (11.5) | 108 (8.8) |  |
| 10 | 1717 (10.6) | 159 (8.3) |  | 588 (10.4) | 48 (7.0) |  | 1129 (10.7) | 111 (9.0) |  |
| *Missing Data* | *3236/19430* | *466/2380* |  | *1179/6826* | *160/841* |  | *2057/12604* | *301/1539* |  |
| Smoking Status (n %) |  |  |  |  |  |  |  |  |  |
| Current | 4160 (21.4) | 622 (26.1) | <0.001 | 1865 (27.3) | 270 (32.1) | <0.001 | 2295 (18.2) | 352 (22.9) | <0.001 |
| Ex-smoker | 12367 (63.7) | 1209 (50.8) |  | 4003 (58.7) | 408 (48.5) |  | 8364 (66.4) | 801 (52.0) |  |
| Never smoked | 2892 (14.9) | 549 (23.1) |  | 954 (14.0) | 163 (19.4) |  | 1938 (15.4) | 386 (25.1) |  |
| *Missing data* | *11 (0.0)* | *0 (0.0)* |  | *4 (0.0)* | *0 (0.0)* |  | *7 (0.0)* | *0 (0.0)* |  |
| Pre-operative Medications n (%) |  |  |  |  |  |  |  |  |  |
| No medications recorded | 784/19430 (4.0) | 109/2380 (4.6) | 0.226 | 298/6826 (4.4) | 40/841 (4.8) | 0.666 | 486/12604 (3.9) | 69/1539 (4.5) | 0.260 |
| Statin | 15652/19430 (80.6) | 1834/2380 (77.1) | <0.001 | 5536/6826 (81.1) | 650/841 (77.3) | 0.009 | 10116/12604 (80.3) | 1184/1539 (76.9) | 0.002 |
| Beta blocker | 5940/19430 (30.6) | 675/2380 (28.4) | 0.029 | 1864/6826 (27.3) | 212/841 (25.2) | 0.211 | 4076/12604 (32.3) | 463/1539 (30.1) | 0.078 |
| ACEi/ARB | 7410/19430 (38.1) | 821/2380 (34.5) | 0.001 | 2522/6826 (36.9) | 291/841 (34.6) | 0.196 | 4888/12604 (38.8) | 530/1539 (34.4) | 0.001 |
| Anticoagulation | 413/19430 (2.1) | 32/2380 (1.3) | 0.014 | 109/6826 (1.6) | 13/841 (1.5) | 1.000 | 304/12604 (2.4) | 19/1539 (1.2) | 0.005 |
| Antiplatelet | 14620/19430 (75.2) | 1719/2380 (72.2) | 0.006 | 5231/6826 (76.6) | 612/841 (72.8) | 0.043 | 9389/12604 (74.5) | 1107/1539 (71.9) | 0.032 |
| Anatomical Factors n (%) |  |  |  |  |  |  |  |  |  |
| AAA diameter, mean (SD), mm | 61.62 (10.16) | 60.13 (9.01) | <0.001 | 62.40 (10.86) | 60.69 (9.69) | <0.001 | 61.20 (9.75) | 59.82 (8.60) | <0.001 |
| Aortic Size Index, mean (SD) | 3.07 (0.62) | 3.46 (0.69) | <0.001 | 3.09 (0.64) | 3.52 (0.73) | <0.001 | 3.05 (0.61) | 3.43 (0.67) | <0.001 |
| Symptomatic | 641/19430 (3.3) | 132/2380 (5.5) | <0.001 | 267/6826 (3.9) | 68/841 (8.1) | <0.001 | 374/12604 (3.0) | 64/1539 (4.2) | 0.014 |
| Neck Angle 0-60 degrees |  |  |  |  |  |  | 11387 (92.3) | 1227 (80.9) | <0.001 |
| Neck Angle 60-75 degrees |  |  |  |  |  |  | 582 (4.7) | 143 (9.4) |  |
| Neck Angle 75-90 degrees |  |  |  |  |  |  | 300 (2.4) | 91 (6.0) |  |
| Neck Angle >90 degrees |  |  |  |  |  |  | 72 (0.6) | 55 (3.6) |  |
| *Neck Angle – Missing Data* |  |  |  |  |  |  | *263/12604* | *23/1539* |  |
| Neck Diameter mean (SD). |  |  |  |  |  |  | 24.22 (4.73) | 22.87 (5.61) | <0.001 |
| Neck Length mean (SD) |  |  |  |  |  |  | 26.72 (14.38) | 24.42 (14.99) | <0.001 |

**Table S2.** Sex-specific differences in receipt of pre-operative assessment and standard of care.

*Abbreviations:, CVD – Cardiovascular Disease, EVAR – Endovascular Aortic Repair, IHD – Ischaemic Heart Disease , IRAAA – Infrarenal Abdominal Aortic Aneurysm, OAR – Open Aortic Repair, p = p value, SD – Standard Deviation.*

|  | All IRAAA | | | OAR | | | EVAR | | |
| --- | --- | --- | --- | --- | --- | --- | --- | --- | --- |
|  | Men | Women | p | Men | Women | p | Men | Women | p |
| n | 19430 | 2380 |  | 6826 | 841 |  | 12604 | 1539 |  |
| Preoperative Assessment n (%) | | | | | | | | | |
| Waiting time mean (SD), days | 61.59 (62.21) | 68.97 (63.64) | <0.001 | 58.95 (62.16) | 76.08 (71.93) | <0.001 | 63.04 (62.20) | 65.03 (58.20) | 0.285 |
| Anaesthetist review | 17392/19430 (91.9) | 2136/2380 (91.0) | 0.163 | 5967/6826 (91.1) | 752 (91.3) | 0.928 | 11425 (92.3) | 1384 (90.9) | 0.061 |
| No fitness tests | 3289/19430 (16.9) | 402/2380 (16.9) | 0.987 | 762/6826 (11.2) | 80/841 (9.5) | 0.166 | 2527/12604 (20.0) | 322/1539 (20.9) | 0.440 |
| Specialty referral | 1236/19430 (6.4) | 146/2380 (6.1) | 0.67 | 433/6826 (6.3) | 66/841 (7.8) | 0.063 | 803/12604 (6.3) | 80/1539 (5.2) | 0.073 |
| Pre-operative Care n (%) | | | | | | | | | |
| General anaesthetic |  |  |  | 6236/6278 (99.3) | 762/771 (98.8) | 0.168 | 9012/12465 (72.3) | 1102/1522 (72.4) | 0.943 |
| Antibiotics | 9007/19430 (46.4) | 1048/2380 (44.0) | 0.034 | 3168/6826 (46.4) | 354/841 (42.1) | 0.020 | 5839/12604 (46.3) | 694/1539 (45.1) | 0.374 |
| DVT Prophylaxis | 8820/19430 (45.4) | 1033/2380 (43.4) | 0.069 | 3083/6826 (45.2) | 346/841 (41.1) | 0.029 | 5737/12604 (45.5) | 687/1539 (44.6) | 0.531 |
| Standard of Care n (%) | | | | | | | | | |
| Pre-op assessment | 14084/19430 (72.5) | 1717/2380 (72.1) | 0.742 | 4887/6826 (71.6) | 613/841 (72.9) | 0.455 | 9197/12604 (73.0) | 1104/1539 (71.7) | 0.319 |
| Waiting time | 9279/15645 (59.3) | 983/1909 (51.5) | <0.001 | 3460/5505 (62.9) | 327/679 (48.2) | *<0.001* | 5819/10051 (57.9) | 656/1230 (53.3) | 0.003 |
| CVD prevention | 9190/19430 (47.3) | 1241/2380 (52.1) | <0.001 | 3669/6826 (53.8) | 467/841 (55.5) | 0.001 | 5521/12604 (43.8) | 774/1539 (50.3) | <0.001 |
| IHD prevention | 2902/7326 (39.6) | 241/691 (34.9) | 0.015 | 893/2094 (42.7) | 76/209 (36.4) | 0.08 | 2009/5232 (38.4) | 165/482 (34.2) | 0.07 |
| Peri-op medications | 8392/19430 (43.2) | 977/2380 (41.1) | 0.049 | 2952/6826 (43.2) | 324/841 (38.5) | 0.010 | 5440/12604 (43.2) | 653/1539 (42.4) | 0.604 |

**Table S3.** Sex-specific differences in post-operative complications

*Abbreviations:, EVAR – Endovascular Aortic Repair, IRAAA – Infrarenal Abdominal Aortic Aneurysm, ITU – Intensive Treatment Unit, MACE – Major Adverse Cardiovascular Event, MACED – Major Adverse Cardiovascular Event or Death, OAR – Open Aortic Repair, p = p value, SD – Standard Deviation.*

|  | IR OAR | | | IR EVAR | | |
| --- | --- | --- | --- | --- | --- | --- |
|  | Men | Women | p | Men | Women | p |
| n | 6826 | 841 |  | 12604 | 1539 |  |
| Major Complications n (%) | | | | | | |
| Death | 194/6826 (2.8) | 37/841 (4.4) | 0.017 | 62/12604 (0.5) | 23/1539 (1.5) | <0.001 |
| MACE | 465/6491 (7.2) | 69/773 (8.9) | <0.001 | 321/12523 (2.6) | 60/1516 (4.0) | <0.001 |
| MACED | 612/6826 (8.6) | 101/841 (12) | <0.001 | 370/12604 (2.9) | 76/1539 (4.9) | <0.001 |
|  | | | | | | |
| ITU stay, mean (SD), days | 3.32 (4.97) | 3.92 (5.98) | 0.003 | 0.44 (1.64) | 0.62 (1.82) | <0.001 |
| Hospital stay, mean (SD), days | 10.92 (18.07) | 13.59 (18.48) | <0.001 | 4.35 (12.23) | 6.11 (19.01) | <0.001 |
| Return to theatre | 443/6818 (6.5) | 63/841 (7.5) | 0.307 | 227/12603 (1.8) | 66/1539 (4.3) | <0.001 |
| Readmission to ITU | 247/6818 (3.6) | 46/841 (5.5) | 0.011 | 97/12603 (0.8) | 25/1538 (1.6) | 0.001 |
| Postoperative Complications | | | | | | |
| Major complication | 1265/6491 (19.5) | 187/773 (24.2) | 0.002 | 754/12523 (6.0) | 137/1516 (9.0) | <0.001 |
| Failure to Rescue | 70/1265 (5.5) | 11/187 (5.9) | 0.981 | 25/754 (3.3) | 14/137 (10.2) | 0.001 |
| Cardiac | 267/6491 (4.1) | 47/773 (6.1) | 0.014 | 204/12523 (1.6) | 28/1516 (1.8) | 0.602 |
| Respiratory | 520/6491 (8.0) | 74/773 (9.6) | 0.153 | 184/12523 (1.5) | 26/1516 (1.7) | 0.527 |
| Stroke | 31/6491 (0.5) | 3/773 (0.4) | 0.948 | 18/12523 (0.1) | 4/1516 (0.3) | 0.440 |
| Renal | 152/6491 (2.3) | 27/773 (3.5) | 0.067 | 95/12523 (0.8) | 17/1516 (1.1) | 0.178 |
| Major Bleeding | 60/6491 (0.9) | 10/773 (1.3) | 0.424 | 85/12523 (0.7) | 16/1516 (1.1) | 0.139 |
| Limb Ischaemia | 124/6491 (1.9) | 11/773 (1.4) | 0.419 | 86/12523 (0.7) | 23/1516 (1.5) | 0.001 |
| Paraplegia | 59/6491 (0.9) | 12/773 (1.6) | 0.127 | 80/12523 (0.6) | 6/1516 (0.4) | 0.331 |
| Bowel Ischaemia | 60/6491 (0.9) | 10/773 (1.3) | 0.424 | 14/12523 (0.1) | 4/1516 (0.3) | 0.237 |

Table S4. Sex specific odds of MACED: (1) unadjusted odds for women followed by forwards stepwise models adjusting for (2) age (≥ 75 years), (3) EVAR, (4) sociodemographic factors (UK deprivation quintile and smoking status), (5) co-morbid status (ischaemic heart disease, congestive heart failure, peripheral arterial disease, chronic kidney disease, stroke, cancer, hypoalbuminaemia (<35 g/dL), anaemia (<13g/dL for men and <12g/dL for women), abnormal ECG, ASA), (6) medications (anticoagulation, betablocker and statin), (7) pre-operative standard of care (pre-operative assessment, treatment within waiting time targets, cardiovascular risk prevention, need for medication adjustment and speciality referral) and (8) AAA repair factors (aortic size index, symptomatic status and general anaesthetic), and (9) within the ‘optimal’ logistic regression model selected by backwards selection.

*Abbreviations: AAA – Abdominal Aortic Aneurysm, American Society of Anaesthesiology, ECG – electrocardiogram, z-value – regression coefficient divided by the standard error, MACED – Major Adverse Cardiovascular Events and/or Death*

| Model | Sequential Addition of Variables | Sex-specific Odds Ratio | z-value | p value | Lower 95% Confidence Interval | Upper 95% Confidence Interval |
| --- | --- | --- | --- | --- | --- | --- |
| 1 | Sex: Woman | 1.51 | 4.86 | <0.001 | 1.27 | 1.78 |
| 2 | Age ≥ 75 years | 1.47 | 4.54 | <0.001 | 1.24 | 1.74 |
| 3 | Repair type | 1.38 | 3.73 | <0.001 | 1.16 | 1.63 |
| 4 | Sociodemographic factors | 1.38 | 3.72 | <0.001 | 1.16 | 1.64 |
| 5 | Co-morbid status | 1.39 | 3.72 | <0.001 | 1.17 | 1.65 |
| 6 | Medications | 1.39 | 3.73 | <0.001 | 1.17 | 1.65 |
| 7 | Standard of Care | 1.36 | 3.42 | <0.001 | 1.14 | 1.61 |
| 8 | AAA repair factors | 1.33 | 3.17 | 0.001 | 1.11 | 1.59 |
| 9 | Optimal' model via backwards selection | 1.35 | 3.43 | 0.001 | 1.14 | 1.61 |

Table S5. ‘Optimal’ logistic regression model selected by backwards selection.

*Abbreviations: AIC – Akaike Information Criteria, ASA - American Society of Anaesthesiology (reference category = ASA 1), COPD – Chronic Obstructive Pulmonary Disease, Deprivation Quintile (reference category = 1), ECG – electrocardiogram, EVAR – Endovascular Aortic Repair, SOC - Standard of care, z-value – regression coefficient divided by the standard error.*

| Coefficients: | Odds Ratio | z-value | p value | Lower 95% Confidence Interval | Upper 95% Confidence Interval |
| --- | --- | --- | --- | --- | --- |
| Sex: Woman | 1.35 | 3.43 | <0.001 | 1.14 | 1.61 |
| Age ≥ 75 years | 1.63 | 7.26 | <0.001 | 1.43 | 1.86 |
| EVAR | 0.26 | -18.80 | <0.001 | 0.23 | 0.30 |
| Deprivation Quintile 3 | 1.13 | 1.43 | 0.153 | 0.95 | 1.34 |
| Deprivation Quintile 4 | 1.13 | 1.51 | 0.132 | 0.96 | 1.33 |
| Deprivation Quintile 5 | 1.20 | 2.17 | 0.030 | 1.02 | 1.41 |
| Current Smoker | 1.12 | 1.50 | 0.135 | 0.97 | 1.30 |
| Ischaemic Heart Disease | 1.14 | 1.87 | 0.062 | 0.99 | 1.31 |
| Congestive Heart Failure | 1.33 | 1.99 | 0.0467 | 1.00 | 1.76 |
| Peripheral Arterial Disease | 2.04 | 3.15 | 0.002 | 1.31 | 3.19 |
| Stroke | 1.24 | 1.85 | 0.064 | 0.99 | 1.56 |
| Anaemia | 1.24 | 2.78 | 0.005 | 1.06 | 1.44 |
| Abnormal ECG | 1.13 | 1.74 | 0.082 | 0.98 | 1.30 |
| ASA Grade 3 | 1.27 | 3.10 | 0.002 | 1.09 | 1.48 |
| ASA Grade 4 | 1.67 | 3.34 | <0.001 | 1.24 | 2.25 |
| COPD | 1.11 | 1.42 | 0.155 | 0.96 | 1.28 |
| BetaBlocker_X1 | 1.12 | 1.64 | 0.100 | 0.98 | 1.29 |
| SOC Preoperative Assessment | 0.86 | -2.32 | 0.021 | 0.75 | 0.98 |
| SOC Waiting Time Treatment | 0.77 | -4.13 | <0.001 | 0.68 | 0.87 |
| Symptomatic Aneurysm | 1.28 | 1.72 | 0.085 | 0.97 | 1.69 |
| General Anaesthetic | 1.31 | 2.40 | 0.017 | 1.05 | 1.63 |
| Null deviance: 9058.2 on 21809 degrees of freedom Residual deviance: 8484.1 on 21788 degrees of freedom AIC: 8528.06  Number of Fisher Scoring iterations: 6 | | | | | |
|  |  |  |  |  |  |

**Table S6.** Sex-specific effect of standard of care on risk of MACED. Note: Interaction P values for pre-operative assessment (p=.175) and waiting time (p=.555) were not significant.

*Abbreviations: z-value – regression coefficient divided by the standard error.*

| Standard of Care Variable | Sex | Odds Ratio | z-value | p value | Lower 95% Confidence Interval | Upper 95% Confidence Interval |
| --- | --- | --- | --- | --- | --- | --- |
| Pre-op Assessment | Man | 0.89 | -1.62 | 0.106 | 0.77 | 1.03 |
| Pre-op Assessment | Woman | 0.69 | -2.23 | 0.026 | 0.50 | 0.97 |
| Waiting Time | Man | 0.76 | -3.89 | <0.001 | 0.67 | 0.87 |
| Waiting Time | Woman | 0.84 | -0.93 | 0.354 | 0.62 | 1.16 |

**Supplementary Appendixes**

**Appendix S1.** Summary of missing data:

(a) percentage of missing data for open repair cohort stratified by sex, (b) percentage of missing data for endovascular repair cohort stratified by sex, (c) Upset plot illustrating combinations and intersections of missingness among variables^1,2^.

**Appendix S2.** Summary of K-nearest neighbours’ multiple imputation: comparison of continuous variables prior to and following multiple imputation.

|  | MEN | | | | WOMEN | | | |
| --- | --- | --- | --- | --- | --- | --- | --- | --- |
|  | 0 | 1 | NA | % | 0 | 1 | NA | % |
| Anaemia | 10529 | 3180 | 5721 | 23.2 | 1265 | 371 | 744 | 22.68 |
| Anaemia_Imp | 15790 | 3640 | 0 | 18.73 | 1947 | 433 | 0 | 18.19 |
| HypoAlbuminaemia | 11734 | 1762 | 5934 | 13.06 | 1398 | 269 | 714 | 16.14 |
| HypoAlbuminaemia_Imp | 17558 | 1872 | 0 | 9.635 | 2093 | 287 | 0 | 12.06 |
| SOCWaitingTime | 6277 | 9279 | 3874 | 59.65 | 926 | 983 | 471 | 51.49 |
| SOCWaitingTime_Imp | 7566 | 11864 | 0 | 61.06 | 1124 | 1256 | 0 | 52.77 |
| GeneralAnaesthetic | 3497 | 15248 | 685 | 81.34 | 429 | 1864 | 87 | 81.29 |
| GeneralAnaesthetic_Imp | 3519 | 15911 | 0 | 81.89 | 436 | 1944 | 0 | 81.68 |
| AbnormalECG | 13043 | 6362 | 25 | 32.79 | 1704 | 667 | 9 | 28.13 |
| AbnormalECG_Imp | 13064 | 6366 | 0 | 32.76 | 1712 | 668 | 0 | 28.07 |

|  | 1 | 2 | 3 | 4 | 5 | NA |
| --- | --- | --- | --- | --- | --- | --- |
| DeprivQuintile_men | 2443 | 3048 | 3547 | 3625 | 3531 | 3236 |
| DeprivQuintile_Imp_men | 2811 | 3628 | 4348 | 4401 | 4242 | 0 |
| DeprivQuintile_women | 397 | 379 | 428 | 389 | 321 | 466 |
| DeprivQuintile_Imp_women | 477 | 471 | 532 | 487 | 413 | 0 |

|  | 1 | 2 | 3 | NA |
| --- | --- | --- | --- | --- |
| SmokingStatus_men | 2892 | 12367 | 4160 | 11 |
| Smoking Status_imp_men | 2893 | 12375 | 4162 | 0 |
| SmokingStatus_women | 549 | 1209 | 622 | 0 |
| Smoking Status_imp_women | 549 | 1209 | 622 | 0 |

|  | 1 | 2 | 3 | 4 | NA |
| --- | --- | --- | --- | --- | --- |
| ASA_men | 198 | 5438 | 13006 | 780 | 8 |
| ASA_Imp_men | 198 | 5439 | 13013 | 780 | 0 |
| ASA_women | 25 | 568 | 1653 | 134 | 0 |
| ASA_Imp_women | 25 | 568 | 1653 | 134 | 0 |

|  | min | Q1 | Median | Mean | Q3Max | Max | NA |
| --- | --- | --- | --- | --- | --- | --- | --- |
| Age_men | 23.8 | 68.8 | 74.3 | 74.34 | 79.8 | 112.3 | 1 |
| Age_Imp_men | 23.8 | 68.8 | 74.3 | 74.24 | 79.8 | 112.3 | 0 |
| Age_women | 29.3 | 72.3 | 77.1 | 76.69 | 81.7 | 93.8 | 1 |
| Age_Imp_women | 29.3 | 72.3 | 77.1 | 76.69 | 81.7 | 93.8 | 0 |
| AaaSize_men | 16 | 56 | 60 | 61.62 | 65 | 200 | 6 |
| AaaSize_Imp_men | 16 | 56 | 60 | 61.62 | 65 | 200 | 0 |
| AaaSize_women | 15 | 55 | 58 | 60.13 | 63 | 120 | 0 |
| AaaSize_Imp_women | 15 | 55 | 58 | 60.13 | 63 | 120 | 0 |
| ASI_men | 0.752 | 2.712 | 2.97 | 3.066 | 3.31 | 11.16 | 105 |
| ASI_Imp_men | 0.752 | 2.713 | 2.972 | 3.066 | 3.31 | 11.16 | 0 |
| ASI_women | 0.941 | 3.037 | 3.372 | 3.464 | 3.797 | 7.606 | 7 |
| ASI_Imp_women | 0.941 | 3.037 | 3.37 | 3.463 | 3.797 | 7.606 | 0 |
| BMI_men | 6.228 | 25.05 | 27.47 | 28.88 | 30.62 | 542.1 | 102 |
| BMI_Imp_men | 6.228 | 25.06 | 27.47 | 28.88 | 30.59 | 542.1 | 0 |
| BMI_women | 10.88 | 23.31 | 26.37 | 28.09 | 30.12 | 841.9 | 7 |
| BMI_Imp_women | 10.88 | 23.31 | 26.37 | 28.08 | 30.11 | 841.9 | 0 |


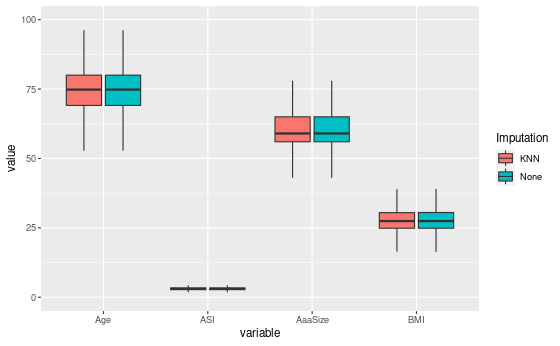


**References**

1 Lex A, Gehlenborg N, Strobelt H, Vuillemot R, Pfister H. UpSet: Visualization of Intersecting Sets. *IEEE Trans Vis Comput Graph*. 2014; **20**: 1983–1992.

2 Conway JR, Lex A, Gehlenborg N. UpSetR: an R package for the visualization of intersecting sets and their properties. *Bioinformatics* [Internet]. 2017 Sep 15; **33**: 2938–2940. Available from: https://doi.org/10.1093/bioinformatics/btx364
